# Effects of High-Biotin Sample Interference on Antibody Concentrations in Sandwich Immunoassays

**DOI:** 10.3390/vaccines11111627

**Published:** 2023-10-24

**Authors:** Geraldo Balieiro Neto, Jair Rodini Engracia Filho, Fabio Enrique Lemos Budino, Acyr Wanderley de Paula Freitas, Weber Vilas Boas Soares

**Affiliations:** 1 Animal Science Institute, Sao Paulo Agency for Agribusiness Technology–APTA, Department of Agriculture and Food Supply, Ribeirao Preto 14030-670, SP, Brazil; fbudino@sp.gov.br (F.E.L.B.); awfreitas@sp.gov.br (A.W.d.P.F.); weber.soares@sp.gov.br (W.V.B.S.); 2 Graduate Program of Animal Science, School of Life Sciences, Pontificia Universidade Catolica do Parana, Curitiba 80215-901, PR, Brazil; jair.rodini@hotmail.com

**Keywords:** antibody–antigen complex, biotin, ELISA, immunoassays, interference, streptavidin

## Abstract

The use of antimicrobial growth promoters (AGPs) is banned because of problems associated with drug residues in animal products and increased bacterial resistance. The immunization of chickens with specific antigens is a promising strategy for generating specific antibodies that can target a wide range of antibiotic-resistant bacteria and can be used as an alternative to antibiotics. Immunoglobulin Y (IgY) antibodies in a polyclonal antibody (pAb) format, when administered orally, modulate the ruminal microbiome and maintain animal health and performance; however, there are concerns pertaining to protein impurities and biotin concentrations in the samples. Signal amplification strategies involving the noncovalent interaction of biotin with streptavidin is extensively used in diagnosis and scientific research, particularly in enzyme-linked immunosorbent assays (ELISAs). However, the high concentrations of biotin in samples, especially in those derived from rich sources such as egg yolk, can pose challenges and potentially harm the accuracy of diagnostic tests and protein concentration measurements. This study aimed to evaluate the influence of biotin on the measurement of IgY in freeze-dried egg yolk samples obtained from immunized laying hens using immunoassays with biotin–avidin/streptavidin. The detection of IgY in yolk samples using ELISA with streptavidin–biotin binding could lead to misdiagnosis due to biotin interference; the level of interference varies with the specific assay conditions and the concentration of biotin in the yolk samples. An ELISA without streptavidin–biotin binding is advisable to avoid interactions between biotin and target proteins, prevent biotin interference with the results, and achieve more reliable and accurate results.

## 1. Introduction

Antimicrobial resistance (AMR) is a global health crisis with significant consequences. Approximately 700,000 fatalities are attributed to AMR annually. By 2050, AMR could lead to approximately 10 million deaths per year and global social costs of USD 100 trillion, underscoring the wide-ranging impact of AMR [1]. The use and misuse of in-feed antibiotics in animal agriculture as growth promoters in poultry and livestock have raised significant concerns related to drug residues in animal products and the development of AMR [2].

There is a growing interest in immunoglobulin Y (IgY), which is abundant in the egg yolks of immunized laying hens, as an alternative to antibiotics for various diagnostic, therapeutic, and research applications [3]. Antibodies against *Streptococcus equinus*, *Lactobacillus* spp., *Fusobacterium necrophorum*, *Escherichia coli*, *Clostridium aminophilum*, *sticklandii*, *Peptostreptococcus*, *Salmonella enteritidis*, *Salmonella enterica*, *Salmonella typhimurium*, *Staphylococcus aureus*, *Helicobacter pylori*, *Pseudomonas aeruginosa*, rotavirus, porcine transmissible gastroenteritis virus, porcine epidemic diarrhea virus, and yeasts have been studied [4,5,6,7,8,9].

IgY antibodies can be obtained in larger quantities from the egg yolks of immunized laying hens compared to those obtained from other traditional sources, such as serum from rats, rabbits, goats, and sheep [10]. Unlike traditional mammalian antibody production, which could involve blood collection that can be stressful, other invasive procedures, and even sacrificing the animal [11], this non-invasive egg collection and IgY extraction from egg yolk is cost-effective and ethical and is a sustainable source of antibodies [12]. Furthermore, egg yolk antibodies do not interact with rheumatoid factors in the serum of mammals and do not bind to proteins A and G, mammalian Fc receptors, or mammalian complements [13]. IgY antibodies are stable and resistant to degradation, allowing for long-term storage and retained activity through different manufacturing steps. Dried IgY batches can maintain their biological activity for several years [14,15].

The oral administration of IgY as an alternative to antibiotics or for microbiome modulation, especially in the polyclonal antibody (pAb) format, often does not require IgY purification for large-scale production. The use of polyethylene glycol (PEG), such as PEG6000, for protein collection from supernatants is a common method for removing fat from samples; however, it can sometimes result in protein impurities and high levels of biotin in the samples, which can be problematic in certain applications [16]. When using IgY without extensive purification, it is important to consider the high level of biotin naturally present in egg yolks, 988–1050 ng/g [17,18], because this could lead to potential biotin interference in assays; it is necessary to consider appropriate strategies to mitigate its impact. Biotin interference is a recognized challenge in immunoassays using streptavidin–biotin-binding interactions [19]; it can affect the accuracy of the results in both research applications and human diagnostics. Approximately 85% of chemiluminescence immunoassays are based on biotin–avidin/streptavidin and are used by more than two-thirds of laboratories in China [20,21]. All laboratories using signal amplification through biotin–streptavidin interactions face the threat of misdiagnosis due to biotin interference caused by excessive biotin consumption [22,23].

This study aimed to evaluate the influence of biotin on IgY quantification in unpurified freeze-dried egg yolk samples obtained from antigen-inoculated hens using biotin–avidin/streptavidin.

## 2. Materials and Methods

### 2.1. Preparation of Antigen

*Streptococcus equinus* was used as an antigen to produce yolk antibodies. The stock culture was thawed and grown on blood plates for 24–48 h at 38 °C under semianaerobic conditions. Colonies were collected by scratching, and they were aseptically transferred to a broth tube containing 2.5 mL of growth medium. The bacteria were grown in a basal medium (39 °C) that contained (per liter) 22 mmol glucose, 1.7 mmol K_2_HPO_4_, 2.1 mmol KH_2_PO_4_, 3.6 mmol (NH_4_)_2_SO_4_, 8.3 mmol NaCl, 0.75 mmol MgSO_4_·7H_2_O, 0.43 mmol CaCl_2_·2H_2_O, 2.8 mmol cysteine hydrochloride, 38 mmol Na_2_CO_3_, 5 mg/mL casamino acids (Difco), 10 mg/mL Trypticase, and 5 g yeast extract. A 1 L borosilicate bottle was used as the container for preparing the growth medium, which was autoclaved for 15 min at 121 °C and at a pressure of 1 atmosphere. A 40% glucose solution was added at room temperature to prevent caramelization. The pH of the medium was adjusted to 6–7 using either 1 M NaOH or 1 M HCl as required.

The turbidity of the culture was checked and compared to the McFarland standards to obtain a final density of 5 × 10^9^ cells/mL, and the culture was transferred to an Erlenmeyer flask containing 50 mL of culture media. When the culture achieved turbidity at 0.5 McFarland, it was transferred to an Erlenmeyer flask with 150 mL of culture media with CO_2_, which was sealed and incubated for 48 h at 38 °C. The homogenized content of the Erlenmeyer flask was filtered through four layers of cheesecloth. The solid in the filter was washed with 0.9% saline, and the total filtrate was transferred to centrifuge containers. The containers with the filtrate were balanced in pairs and centrifuged at 1000× *g* for 10 min at 4 °C. The supernatant was discarded, and the precipitate was resuspended in 150 mL of McDougall’s solution. The content was redistributed in centrifuge containers and centrifuged at 11,250× *g* for 20 min at 4 °C. The supernatant from the second centrifugation was discarded, and the precipitate was transferred to a single container using a spatula and as little deionized water as possible to obtain the final bacterial pellet. The bacterial pellet was resuspended in phosphate-buffered saline (PBS) (pH 7.4) to obtain 5.3 × 10^8^ colony-forming units/mL of *S. equinus* and a turbidity of 0.5 McFarland. To this solution, 4% formaldehyde (18.5%) was added, which was followed by 30% Imject Alum Adjuvant (TermoFisher Scientific, Life Tech Brasil, Itapevi, Sao Paulo, Brazil). The controls were prepared using the same amounts of PBS, formaldehyde, and adjuvant without bacterial pellets. For antigen adsorption, the adjuvant was added slowly, which was followed by constant agitation for 4 h. These immunologic response inductors were transferred to sterile serum bottles, capped, and stored at 4 °C until further use.

### 2.2. Immunization of Hens with Streptococcus Equinus and Preparation of Samples

White Leghorn hens (25-week-old) were divided into two groups. Solution (500 µL), with or without antigen, was injected deeply into the pectoral muscles of each group every 14 days for 56 days. Eggs were collected weekly, broken, and the shells, yolks, and egg whites were separated. The yolk was subjected to freeze drying and delipidation, according to Akita and Nakai [20], to concentrate the IgY and biotin in the aqueous protein fraction. The lyophilized yolk sample (1 g) was weighed in a 15 mL Falcon tube; 6 mL of phosphate-buffered saline (PBS) and 0.210 g of polyethylene glycol (PEG) 6000 were added. The mixture was vortexed for 1 min and incubated at 4 °C with shaking for 10 min. The samples were centrifuged twice at 4 °C, at 10,000 rpm, for 20 min. The precipitate consisted of solids and fat; one fraction of yellow fat floated above the supernatant, and the transparent supernatant in the middle contained biotin and proteins. The supernatant containing protein and biotin was separated from the solids and fat and centrifuged again; the supernatant was collected using a needle and syringe. The protein fraction was filtered using a funnel and paper filter (40 µm) to remove the suspended insoluble solid residue particles and yellow fat and to clarify the sample for the analysis of IgY and biotin using ‘IDK Biotin ELISA,’ manufactured by Immunodiagnostik AG (Bensheim, Germany). Crude IgY from non-immunized hens was used as the control.

### 2.3. Immunoassays Procedures

To evaluate the interference of biotin in the detection of Immunoglobulin Y (IgY, an antibody class found in chicken yolk), two commercial kits for sandwich enzyme-linked immunosorbent assay (ELISA) and two non-commercial plates specific to this study were used. These commercial kits, used for quantitatively detecting IgY in various biological samples for various purposes, come with all the necessary reagents, including antibodies, substrates, and standards, along with detailed instructions for conducting the assay. The capture antibody was added at a concentration of 0.5–4 µg/mL (pre-coated plate), while they usually use detection antibodies at 0.5–1 µg/mL. In these commercial ELISA kits, the detection antibodies are nonspecific; freeze drying makes the proteins and biotin in the sample very concentrated; therefore, a high dilution factor (1:1,000,000) is required to ensure that the sample reading signal is within the values of the standard curves.

**Figure 1:**
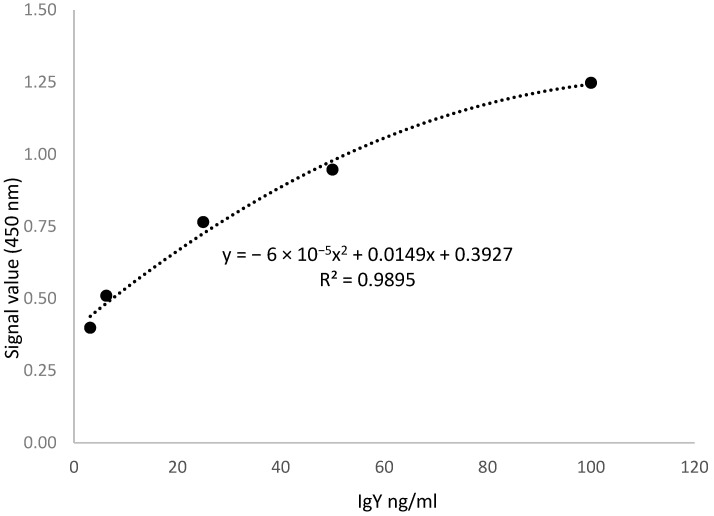
The calibration curve obtained from the serial dilution of the standard from ICH0032 (FineTest).

The commercial test used was ECH0032 from FineTest, in which two specific antibodies were used to “sandwich” IgY between them. One antibody, known as the capture antibody (anti-IgY, usually 0.5–4 µg/mL), was immobilized on a solid surface (e.g., the surface of a microplate well); it binds specifically to IgY. Diluted test samples and standards (0.1 each) were added to the pre-coated plates. The plates were sealed with a cover and incubated at 37 °C for 90 min. The cover was removed, the solution was discarded, and the plate was washed twice with Wash Buffer. Subsequently, 0.1 mL of biotin-conjugated detection antibody was added to the wells, and the plate was sealed with a cover and incubated at 37 °C for 90 min. The cover was removed, and the plate was washed thrice with Wash Buffer; the wash buffer was allowed to stand in the wells for 1 min each time. Horseradish peroxidase (HRP–streptavidin) was added to each well, and the plate was sealed with a cover and incubated at 37 °C for 30 min. Subsequently, the cover was removed, and the plate was washed five times with wash buffer, and the wash buffer was allowed to stand in the wells for 2 min. Then, 90 µL of TMB (3,3′,5,5′ tetramethylbenzidine), the substrate for HRP, was added into each well, and the plate was incubated at 37 °C in the dark for 15–30 min to visualize the HRP enzymatic reaction. Finally, 50 μL of the stop solution was added to each well and mixed thoroughly. The OD_450_ was recorded immediately after adding the stop solution using a spectrophotometer, Biochrom EZ Read 400 Microplate Reader from Holliston, MA, USA. The calibration curve obtained from the serial dilution of the standard is shown in Figure 1.

**Figure 2:**
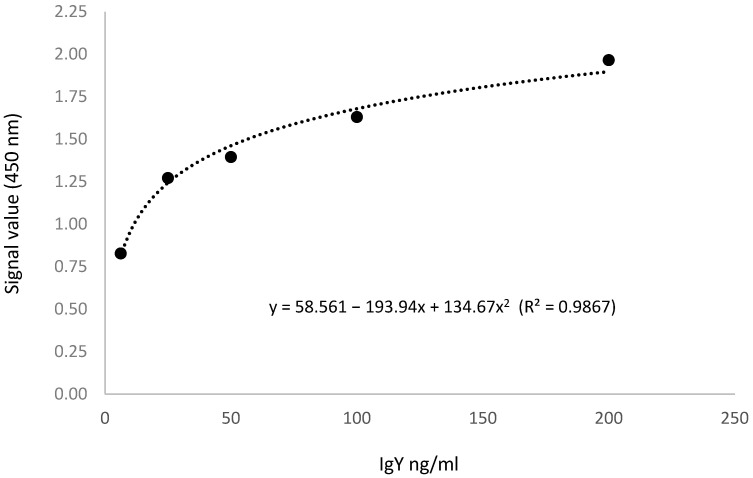
The calibration curve obtained from the serial dilution of the standard from IRKTAH1109.

The IRKTAH1109 test from Innovative Research Incorporation was used to compare the results for IgY concentration obtained through the interaction of biotin with streptavidin without signal amplification. In the IRKTAH1109 assay, the IgY in the samples reacted with the anti-IgY antibodies adsorbed on the surface of polystyrene microtiter wells. After removing the unbound proteins by washing, an enzyme–antibody conjugate (0.1 mL) with horseradish peroxidase (HRP) was added to each well, and these anti-IgY antibodies conjugated with the previously bound IgY to form complexes. The plates were washed, and 100 µL of TMB (3,3′,5,5′ tetra-methylbenzidine), the substrate for HRP, was added into each well; the plates were incubated in the dark at room temperature for 10 min to visualize the HRP enzymatic reaction. Finally, 100 μL of the stop solution was added into each well and mixed thoroughly. The OD_450_ was recorded immediately after the addition of the stop solution using a spectrophotometer. The calibration curve obtained from the serial dilution of the standard is shown in Figure 2.

The interference of biotin in specific antibody detection was evaluated with two-plate trapped antigen-enzyme linked immunosorbent assay (PTA-ELISA). After the bacterial pellet was resuspended in PBS (pH 7.4), the microtiter plate was coated with 100 µL per well of the suspension containing 5.3 × 10^8^ colony-forming units/mL of *S. equinus* using 1 µL/100 µL of 0.05 M carbonate–bicarbonate buffer (Sigma-Aldrich C3041, St. Louis, MO, USA) (pH 9.6) and incubated at 4 °C overnight on plate columns from 3 to 12 (Table 1). The plates were washed thrice with PBS-Tween (PBS-T; 0.85% NaCl in 0.01 M phosphate buffer, pH 7.2, containing 0.05% Tween 20). The plates were treated with cold water fish skin gelatin (0.5%; 200 μL per well) in PBS and incubated at room temperature for 1 h to block the plates. The blocking buffer was removed. Serial dilutions were prepared (Table 1) as follows: 100 µL of PBS was placed into wells (except in columns 1 and 4); 200 µL of sample was placed into the wells of column 4; 100 µL was transferred from wells of column 4 to wells of column 5 (1:1), and then from column 5 to column 6 and so on (1:2; 1:4; 1:8; 1:16; 1:32; 1:64), until column 12 (1:128) (Table 1). The serial dilutions of the samples were performed in duplicate; the plates were sealed with a cover and incubated at 37 °C for 90 min on a soft shaker. The content was discarded, the plates were washed twice, and 100 µL of biotin-labeled antibody was added into the wells. The plates were sealed with a cover and incubated at 37 °C for 60 min. The cover was removed, and the plates were washed thrice; HRP–streptavidin conjugate (0.1 mL) was added to each well, and the plate was covered and incubated at 37 °C for 30 min. Subsequently, the cover was removed, the plate was washed five times with wash buffer, and the wash buffer was allowed to stand in the wells for 2 min. Then, 90 µL of TMB (3,3′,5,5′ tetramethylbenzidine), the substrate for HRP, was added into each well, and the plate was incubated at 37 °C in the dark for 15–30 min to visualize the HRP enzymatic reaction. Finally, 50 μL of the stop solution was added to each well and mixed thoroughly. The OD_450_ was recorded immediately after the addition of the stop solution using a spectrophotometer.

Carbonate–bicarbonate-buffer without antigen (100 µL) was added to the wells of columns 2 and 3 as assay control to monitor direct cross-reactions between the antigen, blocking, target antibodies, biotin, and HRP–streptavidin interactions; the samples were not incubated (Table 1).

Another PTA-ELISA with a specific antigen coated on the wells was used similarly, without signal amplification, through the interaction of biotin with streptavidin. After removing the unbound antibodies by washing, HRP-conjugated rabbit anti-chicken IgY (IgG) (Sigma-Aldrich A9046) was added, which was followed by the same washing steps. Then, 90 µL of TMB (3,3′,5,5′ tetramethylbenzidine), the substrate for HRP, was added into each well, and the plate was incubated at 37 °C in the dark for 15–30 min to visualize the HRP enzymatic reaction. Finally, 50 μL of the stop solution was added to each well and mixed thoroughly. The OD_450_ was recorded immediately after the addition of the stop solution using a spectrophotometer. The intensity of the yellow color was proportional to the concentration of IgY captured from the sample.

### 2.4. Statistical Analysis

The standards that come with commercial plates for quantitative analysis do not contain specific antibodies against the antigens used for coating the wells in the non-commercial plates. We analyzed two samples, from immunized hens and from non-immunized hens, and 5 plates, 3 plates for signal-based detection and 2 commercial plates for IgY concentration. We used the complete randomized block design with a 2 × 3 factorial arrangement (3 plates × 2 samples) for signal values and a 2 × 2 factorial arrangement (2 plates × 2 samples) for IgY concentrations with eight replicates. Regression analysis of the serial dilution assays from PTA-ELISA with streptavidin–biotin detection was performed to fit a better model. Statistical analyses were performed using the GLM procedure of SAS 9.4 (SAS Institute Inc., Cary, NC, USA). The 95th percentile was calculated; *p* < 0.05 was considered statistically significant. The least-square means were adjusted for multiple comparisons using the Tukey–Kramer method.

## 3. Results

**Figure 3:**
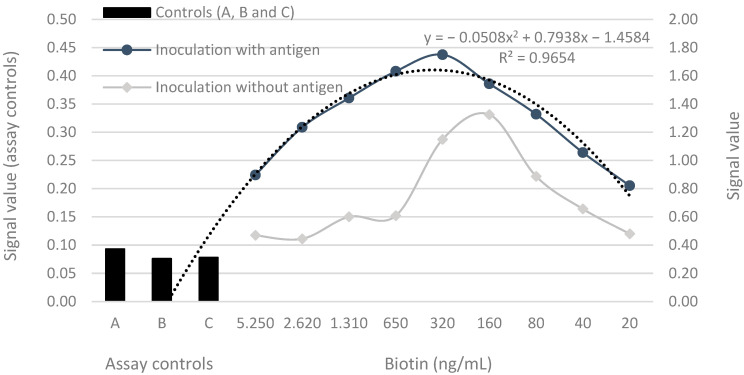
Correlation between the optical signal value for IgY detection (absorbances at 450 nm) and the levels in the serial dilutions in PTA-ELISA. Assay controls: A = without antigen with sample; B = without antigen and sample; and C = with antigen without sample.

The signals for the yolk samples obtained from hens inoculated with the antigen were higher than those for samples from hens inoculated without the antigen (*p* < 0.001). Regression analysis of the serial dilution assays from PTA-ELISA with streptavidin–biotin detection described the relationship between the observed signal and the dilution levels, indicating a significant and stronger relationship in the samples from hens inoculated with the antigen (R^2^ = 0.96) compared to that in samples from hens inoculated without the antigen (R^2^ = 0.52) (Figure 3). The signal values obtained from control wells demonstrated that the blocking worked well; when the antibodies were not bound to the plate without antigen, there was no cross-reaction between the blocking and secondary antibodies or between the secondary antibodies and the antigen pre-coated on the plate (Figure 3).

**Figure 4:**
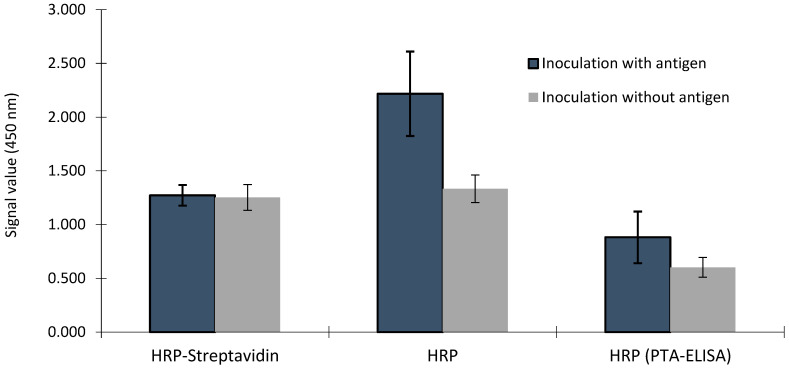
Comparison of IgY detection in yolk samples from hens inoculated with or without antigen through ELISA using streptavidin–biotin binding (HRP-Streptavidin) or not (HRP) and on specific antigen pre-coated plates without the use of streptavidin–biotin binding—HRP (PTA-ELISA).

**Figure 5:**
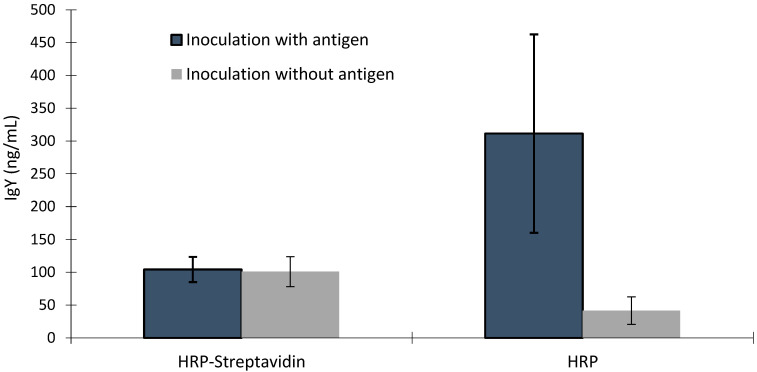
Comparison of IgY concentration in yolk samples from hens inoculated with or without antigen through ELISA using streptavidin–biotin binding (HRP-Streptavidin) or not (HRP).

There was an interaction between the samples from hens inoculated with or without the antigen and the plates containing biotin–avidin/streptavidin (*p* < 0.001). There was no difference in signal values or IgY concentration between samples from hens inoculated with or without antigen in immunoassays using biotin–avidin/streptavidin; however, the signal values and IgY concentrations of the samples from hens inoculated with antigen were higher than that in the samples from hens inoculated without antigen (Figure 4 and Figure 5). There was a trend of higher signal values in the samples from hens inoculated with antigen compared to that in samples from hens inoculated without antigen in the PTA-ELISA without the use of biotin–avidin/streptavidin (*p* < 0.06). The signal values in this plate were lower than those obtained from the commercial ELISA kits (*p* < 0.001), except for those signals obtained in the control sample, in the plate using biotin–avidin/streptavidin.

## 4. Discussion

Various sample components can lead to false positive or false negative results and affect the sensitivity and specificity of an assay. Lipids are components that contribute to the matrix effect; therefore, they are removed. The antibodies pre-coated on the plate were specific anti-IgY antibodies, and the matrix effect was mitigated to a certain extent through delipidation. However, we cannot exclude the matrix effect and the cross-reactions with proteins other than IgY; the high biotin level was likely the main influence in the sample matrix.

The amount of biotin can interfere with complex formation and influence the solubility and availability of Ab in different dilutions and the Ag–Ab relationships. Dilutions decrease the target protein concentrations; therefore, the lower signal values at the initial points of the serial dilutions were unexpected and are one of the most difficult types of interference to explain.

In case of a high concentration of IgY in the sample, a limited number of anti-IgY or antigen sites (Ag) are encountered by a huge number of analyte molecules, resulting in a deficit of anti-IgY or Ag in the solid phase because the anti-IgY or Ag used in the assay can be completely coated and saturated with the analyte. Therefore, the excess antibodies in the sample do not form complexes with anti-IgY or Ag (prozone) because the analyte is only partially bound. This underestimates the IgY concentrations in the samples from immunized hens, generating false similarities between the antibody concentrations of the different treatments.

Undiluted and diluted samples are often tested to detect the prozone effect. If the results of the diluted sample are higher than that of the undiluted sample, then the undiluted sample most likely exhibits the prozone effect. This phenomenon can lead to misleading test results, including false negatives and artificially elevated signals after sample dilution [24]. This could be overcome by adequately diluting the samples; the analyte concentration can be reduced to a level where the anti-IgY or Ag is not saturated, allowing for accurate measurement.

The dilution curve obtained from serial dilutions (Figure 3) is essential for determining the optimal relationship between antigen (Ag) and antibody (Ab) concentrations, and it helps to identify the point where complex formation is most effective. Beyond this point, further dilution reduces the concentration of specific antibodies available for complex formation, leading to a decrease in the signal. The initial dilutions can lead to an upward curve in the assay results owing to the prozone phenomenon and the greater solubility, availability, and dispersion of IgY proteins in the solution, which promotes better contact between the antibodies and the antigen, leading to increased complex formation.

Considering the use of streptavidin–biotin binding as part of the assay methodology, the hypothesis of interference from high amounts of biotin in the sample prevails. When the concentration of biotin in the sample is extremely high, the available binding sites on the reagents can become saturated. This excessive binding can prevent the formation of secondary biotin-labeled antibody complexes, which are washed away before the addition of HRP–streptavidin. This could decrease the number of biotin marker-binding sites in the secondary antibody compared to that in the primary antibody, keeping these sites occupied. In addition, the target antibodies (Ab) in the sample are bound to endogenous biotin even before being captured by the coated anti-IgY and will form streptavidin–biotin complexes, amplifying the signal after the addition of HRP–streptavidin. Therefore, changes in the measured values of sandwich immunoassays can lead to false low or high values in the measured results, depending on the concentrations and the relationships among the proteins, biotin, and reagents in the assays using biotin–avidin/streptavidin. Kabiri et al. [22] studied biotin interference in protein measurements and concluded that biotin in the sample leads to false high values in sandwich immunoassays. Liu et al. [23] observed that biotin in the sample led to a false negative result because the assay failed to detect the target Ab even though it was present in the sample.

Liu et al. [23] tested the levels of streptavidin-coated magnetic microparticles required to neutralize a high concentration of biotin in a sample; the results varied with the biotin and target protein concentration relationships. The results from this study are in line with those from Liu et al. [23] until a dilution factor of 1:16; lower sample dilutions do not result in higher immunocomplex formations. Instead, there was a decrease in biotin interference at higher dilutions, being the amount of IgY on samples from hens inoculated with antigen, enough to increase immunocomplex formations with less biotin interference (Figure 3).

The smaller IgY concentration in the control samples from hens inoculated without antigen was not sufficient to obtain the signal until a dilution factor of 1:16; immediately after the reaction, some nonspecific Abs were bound to the antigen (Figure 3). The strong affinity of streptavidin for biotin can lead to signal amplification even when small amounts of endogenous biotin are present in the sample. This can result in false-positive signals in assays because streptavidin may bind to endogenous biotin, producing a signal that is not related to the target of interest. Therefore, appropriate blocking methods and controls must be employed to address this issue. In some cases, when primary antibodies are not adequately washed off or blocked, streptavidin can amplify signals from nonspecifically bound primary antibodies. This can lead to false positive results or increased background signals. Cross-reactivity with irrelevant antigenic targets is common because of the multiple-epitope recognition of pAbs, which bind to common antigenic motifs [25].

In line with our results, Liu et al. [26] and Samarasinghe et al. [27] found falsely decreased signal values due to biotin interference in sandwich immunoassays. Liu et al. [23] argued that the biotin in the sample and the reagent compete for binding sites on streptavidin reagents; as a result, luminescent substances cannot be captured by streptavidin. They used streptavidin-coated magnetic microparticles to neutralize a high concentration of biotin in a sample; the signals were markedly greater when the concentration of biotin was approximately 500 ng/mL and 1000 ng/mL; the detection results differed by approximately 10% and > 20%, respectively, from those obtained in samples without biotin addition. Similarly, IgY detection increased with a reduction in biotin concentration from 5.250, 2.620, 1.310, and 650 ng/mL to 320 ng/mL, after which biotin interference did not occur, and dilutions began to decrease IgY detection (Figure 3).

If the amount of streptavidin is sufficient for all Abs, when biotin–streptavidin binding is used as part of a ‘one-step sandwich’ immunoassay format, excess biotin in the sample can block the binding of biotinylated antibodies to biotin-binding sites on the streptavidin-coated solid phase, resulting in false low results [19]. However, using a sandwich immunoassay with washing steps, the absence of a difference between the signals of samples from hens inoculated with or without the antigen on plates using biotin–avidin/streptavidin (Figure 4) can be attributed to the same amount of streptavidin being deposited in all wells during the assay, resulting in similar signals even with different IgY concentrations. Avidin and other biotin-binding proteins, including streptavidin, have the ability to bind up to four biotin molecules; however, the reason for biotin interference in samples with high biotin concentrations could be an insufficiency of streptavidin; a case where all added streptavidin binding sites are occupied, making it impossible to differentiate between samples with different Ab concentrations (Figure 4).

A method without signal amplification, using the interaction of biotin with streptavidin, provided clearer differentiation between samples. When signal amplification is not involved, the signal intensity directly reflects the concentration of the target analyte, making it easier to determine the relative differences between samples (Figure 4). Without signal amplification or interference from biotin, plates coated with nonspecific anti-IgY antibodies and plates coated with specific antigens showed similar relative differences between samples for both signal and IgY concentrations (Figure 4 and Figure 5). This suggests that the plates provide consistent results, which is a positive aspect of the assay. The HRP–streptavidin method provided similar IgY concentrations between samples with and without antigen (Figure 5); this combines the issues, leading to the increase and decrease in IgY concentrations, respectively, compromising the comparison between different treatments. The variation observed in the IgY levels in the ELISA assay, where hens inoculated with antigen were tested without employing streptavidin–biotin binding, can be attributed to the immune responses of the hens to antigen inoculation, especially when adjuvants were involved. This could lead to differences in the concentration and specificity of the antibodies produced. However, the IgY concentrations were consistently higher in hens inoculated with antigens compared to that in those inoculated without antigens, particularly in the absence of biotin interference.

The lower signal values of samples from hens inoculated with antigen in the PTA-ELISA compared to their corresponding signals from commercial ELISA kits could be attributed to the measurement of specific Abs; other nonspecific Abs may not bind to the antigen pre-coated on the plate. Egg yolk can provide 70–150 mg of Ab, with only 2–10% Ag specificity for the inoculated antigens [28]. The IgY concentrations in this study were similar to those reported earlier. Losch et al. [29] described that one egg yolk provides 40–500 mg of IgY, while Kowlaczyk et al. [30] showed the amount of IgY in the egg yolk was 15.7 mg/mL with a range from 5.3 to 43.3 mg/mL; in theory, 120 mg IgY can be obtained from one egg. However, there is a wide range of results. The results of the samples with excess biotin, analyzed using ELISA with signal amplification using the biotin–streptavidin interaction, could lead to misdiagnosis owing to biotin interference. Therefore, biotin-stripping methods can be used to mitigate the risk of false negatives and false positives to remove excess biotin from the sample. Biotin-blocking reagents can prevent biotin interference by binding free biotin molecules in the sample before performing the ELISA. An alternative antibody-based detection method that does not involve biotin in the streptavidin–biotin binding can be used for better accuracy.

## 5. Conclusions

The strong affinity of streptavidin for biotin can significantly affect the IgY concentration in different assays, particularly when different levels of antibodies and biotin are present in the sample. Strategies to neutralize biotin levels in samples or alternative detection methods are crucial for avoiding potential biotin interference and ensuring reliable results. Detection methods based on the direct labeling of antibodies with enzymes that do not rely on streptavidin–biotin interactions are desirable and should preferred for preventing biotin interference.

## Figures and Tables

**Table 1:** Serial dilution and the concentrations of biotin and assay controls in the PTA-ELISA columns.

Plate Columns											
01	02	03	04	05	06	07	08	09	10	11	12
Assay controls			Serial dilution (biotin ng/mL)								
no-antigen sample	no-antigen no-sample	Antigen no-sample	5250	2620	1310	650	320	160	80	40	20

## Data Availability

The datasets generated and/or analyzed in the current study are available from the corresponding author upon reasonable request.
